# Genomic and Immunophenotypic Landscape of Acquired Resistance to PD-(L)1 Blockade in Non–Small-Cell Lung Cancer

**DOI:** 10.1200/JCO.23.00580

**Published:** 2024-01-11

**Authors:** Biagio Ricciuti, Giuseppe Lamberti, Sreekar R. Puchala, Navin R. Mahadevan, Jia-Ren Lin, Joao V. Alessi, Alexander Chowdhury, Yvonne Y. Li, Xinan Wang, Liam Spurr, Federica Pecci, Alessandro Di Federico, Deepti Venkatraman, Adriana P. Barrichello, Malini Gandhi, Victor R. Vaz, Andy J. Pangilinan, Danielle Haradon, Elinton Lee, Hersh Gupta, Kathleen L. Pfaff, Emma L. Welsh, Mizuki Nishino, Andrew D. Cherniack, Bruce E. Johnson, Jason L Weirather, Ian D Dryg, Scott J. Rodig, Lynette M. Sholl, Peter Sorger, Sandro Santagata, Renato Umeton, Mark M. Awad

**Affiliations:** ^1^ Lowe Center for Thoracic Oncology, Dana-Farber Cancer Institute, Boston, MA; ^2^ Department of Informatics and Analytics, Dana-Farber Cancer Institute, Boston, MA; ^3^ Department of Pathology, Brigham and Women's Hospital, Boston, MA; ^4^ Laboratory of Systems Pharmacology, Department of Systems Biology, Harvard Medical School, Boston, MA; ^5^ Ludwig Center at Harvard, Harvard Medical School, Boston, MA; ^6^ Cancer Program, Broad Institute of MIT and Harvard, Cambridge, MA; ^7^ Harvard School of Public Health, Boston, MA; ^8^ Center for Immuno-Oncology, Dana-Farber Cancer Institute, Boston, MA; ^9^ Department of Radiology, Brigham and Women's Hospital, Boston, MA

## Abstract

**PURPOSE:**

Although immune checkpoint inhibitors (ICI) have extended survival in patients with non–small-cell lung cancer (NSCLC), acquired resistance (AR) to ICI frequently develops after an initial benefit. However, the mechanisms of AR to ICI in NSCLC are largely unknown.

**METHODS:**

Comprehensive tumor genomic profiling, machine learning–based assessment of tumor-infiltrating lymphocytes, multiplexed immunofluorescence, and/or HLA-I immunohistochemistry (IHC) were performed on matched pre- and post-ICI tumor biopsies from patients with NSCLC treated with ICI at the Dana-Farber Cancer Institute who developed AR to ICI. Two additional cohorts of patients with intervening chemotherapy or targeted therapies between biopsies were included as controls.

**RESULTS:**

We performed comprehensive genomic profiling and immunophenotypic characterization on samples from 82 patients with NSCLC and matched pre- and post-ICI biopsies and compared findings with a control cohort of patients with non-ICI intervening therapies between biopsies (chemotherapy, N = 32; targeted therapies, N = 89; both, N = 17). Putative resistance mutations were identified in 27.8% of immunotherapy-treated cases and included acquired loss-of-function mutations in *STK11*, *B2M*, *APC*, *MTOR*, *KEAP1*, and *JAK1*/*2*; these acquired alterations were not observed in the control groups. Immunophenotyping of matched pre- and post-ICI samples demonstrated significant decreases in intratumoral lymphocytes, CD3e^+^ and CD8a^+^ T cells, and PD-L1–PD1 engagement, as well as increased distance between tumor cells and CD8^+^PD-1^+^ T cells. There was a significant decrease in HLA class I expression in the immunotherapy cohort at the time of AR compared with the chemotherapy (*P* = .005) and the targeted therapy (*P* = .01) cohorts.

**CONCLUSION:**

These findings highlight the genomic and immunophenotypic heterogeneity of ICI resistance in NSCLC, which will need to be considered when developing novel therapeutic strategies aimed at overcoming resistance.

## Teaser

Genomic and immunophenotypic changes shape acquired resistance to #ICI in #NSCLC à Link to the paper @BiagioRicciutMD @GLambertiMD @DanaFarber #LCSM

## INTRODUCTION

Immune checkpoint inhibitors (ICI) produce durable responses and extend survival in patients with advanced non–small-cell lung cancer (NSCLC).^1^ Unfortunately, most responders to ICI will eventually develop disease progression. Although serial tumor genomic profiling has led to the identification of resistance mechanisms to targeted therapies in subsets of lung cancer such as those with *EGFR* mutations or *ALK* rearrangements,^2^ the landscape of acquired resistance (AR) to ICI in NSCLC remains unknown. Recent reports have identified defects in interferon gamma (INFγ) signaling and impaired HLA class I antigen presentation as potential mechanisms of AR to ICI in solid tumors.^3,4^ However, these studies were limited by the retrospective design, small sample sizes, and the lack of a control group. Although rational strategies have been developed to overcome resistance to targeted therapies, there are no approved immunotherapies to overcome resistance to ICI in NSCLC. Thus, understanding the mechanisms of AR to immunotherapy is essential for the development of novel strategies tailored to overcoming specific resistance mechanisms. Here, we analyzed a large cohort of patients with NSCLC and matched pre- and post-ICI samples to characterize the genomic and immunophenotypic landscape of AR to PD-(L)1 inhibition.

## METHODS

Detailed methods, including statistical analysis and methods used for genomic, immunophenotypic, and machine learning (ML) analysis, are reported in the Supplementary Methods.

### Patient Population

Patients with NSCLC who had consented to correlative research protocols at the Dana-Farber Cancer Institute, received treatment with ICI, and developed AR between two repeat biopsies were included. Patients who received other systemic therapies including targeted therapies or chemotherapy were included as controls. AR to systemic treatments was defined as the development of disease progression after an objective response (either partial response [PR] or complete response [CR]) or stable disease (SD) for ≥3 months.

### Immunohistochemistry

The PD-L1 tumor proportion score, beta-2-microglobulin (B2M), and HLA class I expression were assessed with immunohistochemistry (IHC) using validated monoclonal antibodies as summarized in the Supplementary Methods.

### Tumor Genomic Profiling

Targeted exome next-generation sequencing was performed using the validated OncoPanel assay, as previously described.^5^ Tumor mutational burden (TMB) was determined using OncoPanel.^3^

For each gene, the absolute copy number was estimated based on the tumor purity (p) and the weighted average of segmented log_2_ ratios across the gene (l) using the formula

$$ACN=\frac{2^{\left(I+1\right)}-2\left(1-p\right)}{p}$$

To quantify aneuploidy levels and aneuploidy score, targeted sequencing data were analyzed using ASCETS (Arm-level Somatic Copy-number Events in Targeted Sequencing), as previously described (Supplementary Methods).^6,7^

### Tumor Similarity Score

To assess similarity between tumor pairs and determine which pairs arose from shared lineage, a score was computed using an internal pipeline on the basis of the frequency (f_event_) of each single-nucleotide variant, small insertion/deletion, gene-level copy-number alteration (CNA), and structural variant in our NSCLC cohort and p_event_, computed as the square of f_event_, representing the likelihood of observing the event independently in two samples. The formula for similarity score computation for a tumor pair is outlined below, where s = sample and f_i_ = frequency of event i in the cohort.

$${for\;s}_{a},s_{b}\in \left\{s_{1}\ldots s_{n}\right\}:$$

$$Similarity\;score=-\log\left(\overset{m_{events}}{\underset{i=1}{\prod }}\begin{array}{c} if\;{event}_{i}{present\;in\;s}_{a}⋀s_{b},{\alpha *f_{i}}^{2}\\ else,1-{f_{i}}^{2} \end{array}\right)$$

Samples deemed to arise from shared lineage were included in the analysis.

### Multiplexed immunofluorescence

Multiplexed immunofluorescence was performed on histologic tissue samples by staining 5-micron formalin-fixed, paraffin-embedded whole-tissue sections with standard, primary antibodies, as previously described (Supplementary Methods).^8-10^ To examine the interactions between PD-L1 and PD-1 in single-cell data, a k-nearest searching algorithm was used to identify cell neighbors within a 20-micron radius. PD-L1+ cells located in proximity to PD-1+ cells were labeled as PD-1+ interactors. To quantify the PD-1–PD-L1 interaction, all potential PD-1–PD-L1 interaction pairs were normalized by total PD-L1+ cells in each sample.

### ML Assessment of Tumor-Associated Immune Cells

Whole-slide images were converted into tiles of size 2,048 × 2,048 pixels using PathML, as previously described.^11^ Model inference was carried out to identify the following cell types: lymphocytes, epithelial cells, macrophages, and neutrophils. K-nearest neighbor minimum spanning tree graphs, on the basis of spatial proximity, were generated for each cell type using the centroid locations (Supplementary Methods).

### Statistical Analysis

Categorical and continuous variables were summarized using descriptive statistics. The Wilcoxon signed-rank test was used to test for a difference in median of paired observations, and Fisher's exact test or χ^2^ was used to test for associations between categorical variables (Supplementary Methods). All statistical analyses were performed using R version 3.6.1.

## RESULTS

### Patient Characteristics

The schema for this study is shown in the Data Supplement (Fig S1, online only). Among 1,757 patients with NSCLC who received ICI at Dana-Farber Cancer Institute, 1,498 had progressed at the data lock, with a median follow-up of 37.4 months (95% CI, 35.0 to 39.9). Of these, 742 (49.5%) patients developed AR to immunotherapy, defined as an objective response or SD for at least 3 months followed by disease progression, of whom 461 (62.1%) had AR after 6 months of treatment. The cumulative risk of AR over time among all-comers with an objective response or SD ≥3 months is shown in the Data Supplement (Fig S2). Among patients with AR, 82 had matched pre- and post-ICI tissue available for correlative analysis (Data Supplement, Table S1). At the time of AR, 25 (30.5%) experienced systemic progression, while 57 (69.5%) had oligoprogression, defined as disease progression in ≤3 metastatic sites. In this cohort, the median age of patients was 65 years (range, 24-81), 57.3% were women, 79.3% had a history of tobacco use, and 86.6% had adenocarcinoma histology. Driver mutations in *KRAS* were identified in 34.1% of cases. Immunotherapies received were PD-(L)1 monotherapy (56.1%), combined PD-1 and cytotoxic T-cell lymphocyte-4 (CTLA-4) blockade (6.1%), and chemoimmunotherapy (37.8%). Importantly, among these patients, 74.4% received a PD-(L)1–containing regimen as the only intervening therapy between the tumor biopsies. Immunotherapy was given as first line in 59.8% patients and as ≥second line in 40.2% of cases.

In this cohort, 2.4% of patients had a CR, 41.5% had a PR, and 56.1% had SD ≥3 months as the best response to treatment (Data Supplement, Fig 3A). The median progression-free survival (PFS) and overall survival (OS) were 7.9 months (95% CI, 6.8 to 10.4) and 35.0 months (95% CI, 24.7 to 45.1), respectively (Data Supplement, Figs 3B-3C). The median OS from the time of AR and from the post-ICI biopsy was 17.3 months (95% CI, 12.9 to 33.3) and 10.9 months (95% CI, 6.9 to 14.8), respectively (Data Supplement, Fig S4), and the median time between pre- and post-ICI tumor biopsies was 18.9 months. Among the 82 patients with AR to ICI, pre- and post-ICI correlative analyses were available as detailed in the Data Supplement (Table S2 and Fig S5).

Two additional cohorts including a total of 138 patients with NSCLC who had matched tumor biopsies pre- and post-chemotherapy (N = 32), pre- and post-targeted therapies (N = 89), or both (N = 17) were included as controls (Data Supplement, Table S2). The median time between pre- and post-treatment tumor biopsies was 19.8 months in the chemotherapy cohort and 17.7 months in the targeted therapy cohort. Baseline clinicopathologic characteristics of these patients are summarized in the Data Supplement (Table S3). The biopsy site and the correlative analyses performed on each tumor biopsy in patients who received immunotherapy, targeted therapies, and chemotherapy are summarized in the Data Supplement (Table S4). In each of these three cohorts, the tumor purity was similar between pre- and post-treatment tumor samples (Data Supplement, Fig S6).

### Genomic Correlates of AR to PD-(L)1 Blockade in NSCLC

**FIG 1.**
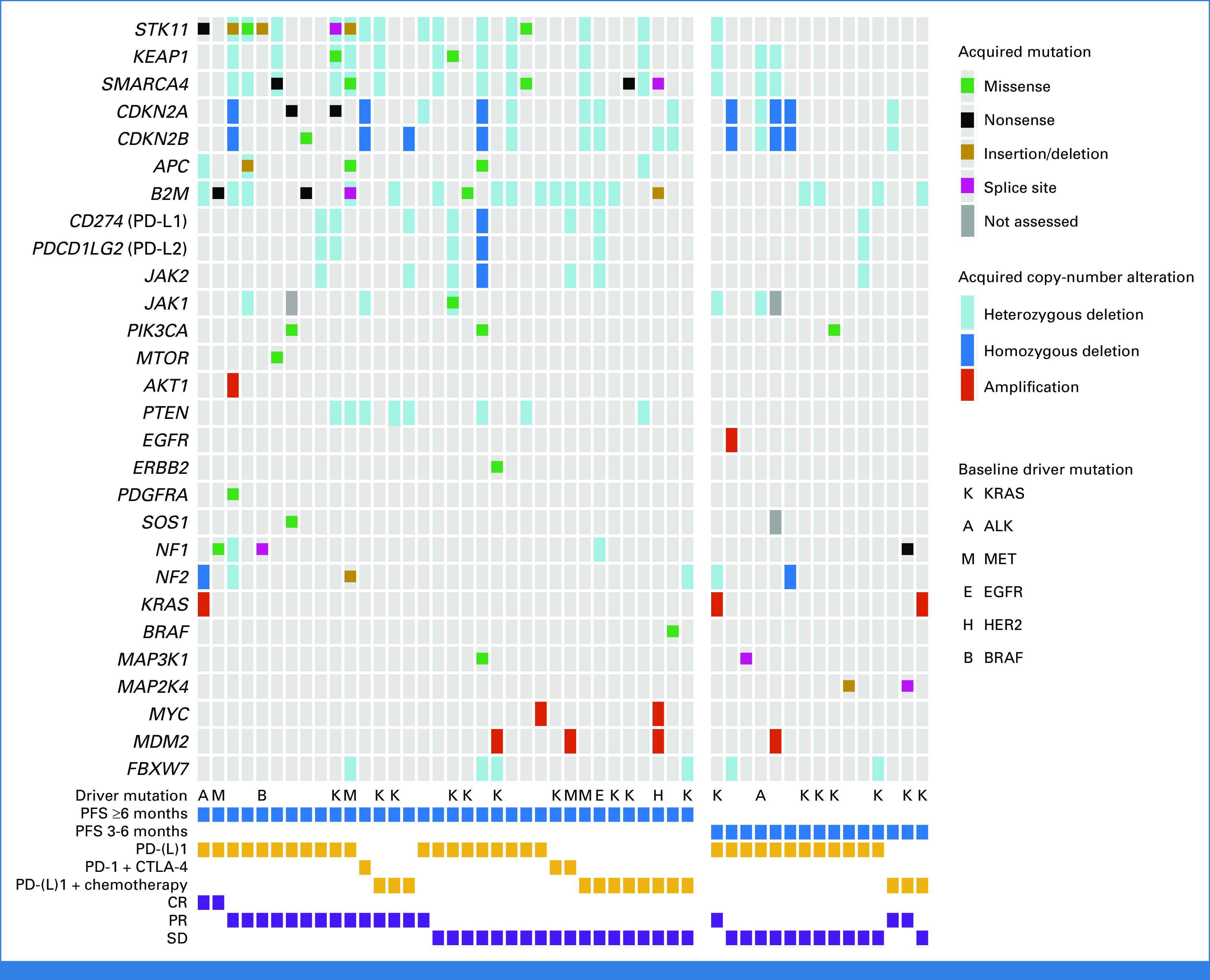
Summary of the genomic and immunophenotypic changes identified at the time of acquired resistance to PD-(L)1–based therapies in patients with NSCLC whose tumor underwent comprehensive genomic profiling at the DFCI. Only acquired genomic alterations in the post-ICI biopsy that were not present in the pre-ICI biopsy are displayed. Samples without acquired genomic changes at the time of resistance are not shown. Driver mutations shown in the oncoprint represent mutations identified at baseline, before the start of immunotherapy. Variants predicted to be benign or originating from clonal hematopoiesis of indeterminate potential are not shown. CR, complete response; CTLA-4, cytotoxic T-cell lymphocyte-4; DFCI, Dana-Farber Cancer Institute; ICI, immune checkpoint inhibitor; NSCLC, non–small-cell lung cancer; PFS, progression-free survival; PR, partial response; SD, stable disease.

**FIG 2.**
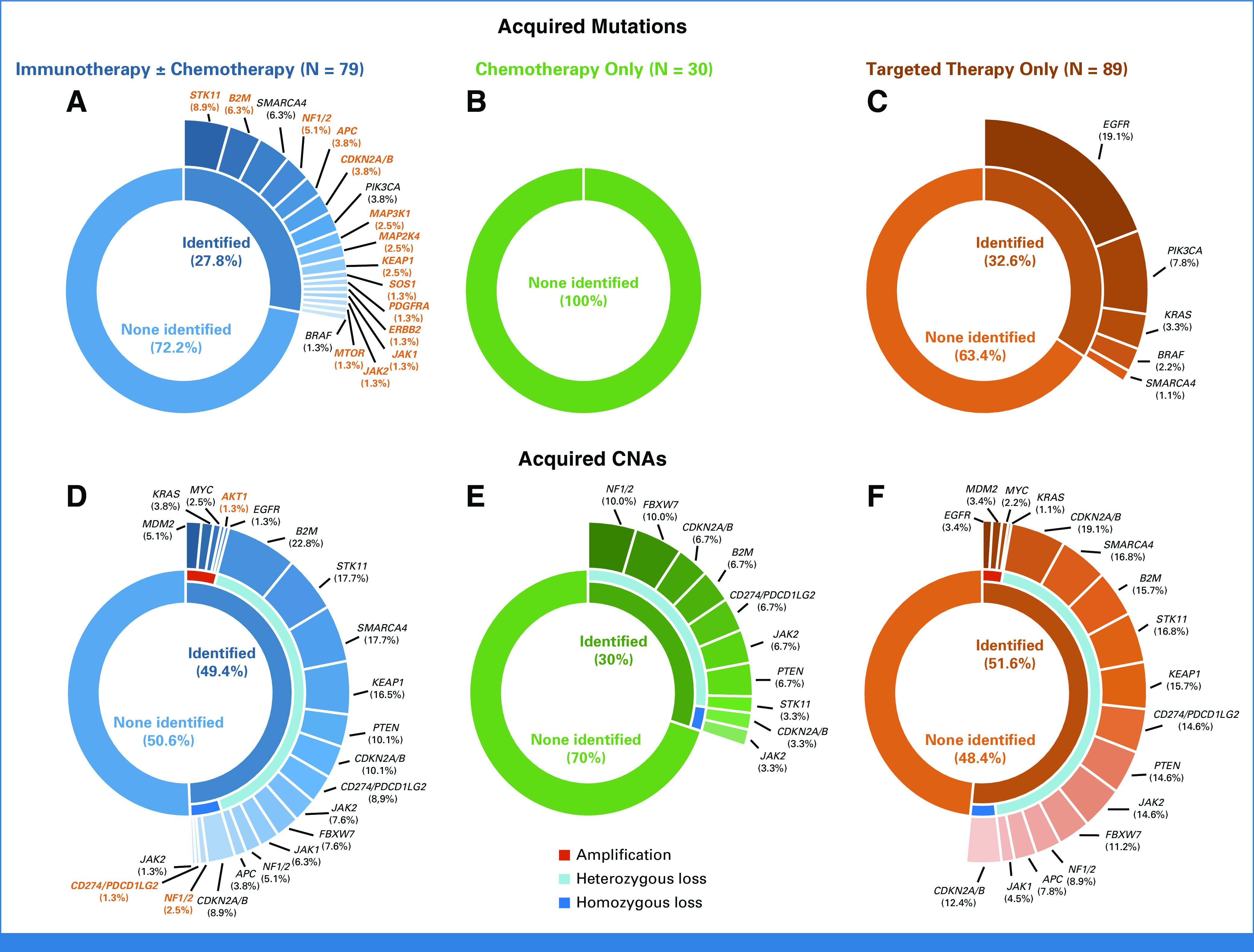
Pie-donut plots depicting the rate of acquired mutation at the time of resistance to (A) PD-(L)1–based therapies, (B) chemotherapy, and (C) targeted therapy in patients with NSCLC and matched pre- and post-treatment tumor genomic profiling. Pie-donut plots depicting the rate of acquired CNAs at the time of resistance to (D) PD-(L)1–based therapies, (E) chemotherapy, and (F) targeted therapy. Labels of genomic alterations uniquely identified in the PD-(L)1–based therapies cohort are in orange. CNA, copy-number alteration; NSCLC, non–small-cell lung cancer.

Genomic changes at the time of AR to ICI were identified in 62.0% (49/79) of patients with pre- and post-ICI tumor genomic profiling (Fig 1). Acquired mutations were identified in 27.8% of cases, while acquired CNAs were found in 49.4% of cases. The frequency of genomic changes identified at the time of resistance by the type of immunotherapy received is shown in the Data Supplement (Fig S7). Among patients with acquired mutations, the most common included loss-of-function alterations in *STK11* (8.9%), *B2M* (6.3%), *SMARCA4* (6.3%), *NF1*/*2* (5.1%), *APC* (3.8%), *CDKN2A*/*B* (3.8%), *MAP3K1* (2.5%), *MAP2K4* (2.5%), *KEAP1* (2.5%), MTOR (1.3%), *JAK1* (1.3%), and *JAK2* (1.3%; Fig 2A). Acquired activating mutations at the time of AR to immunotherapies included *PIK3CA* (3.8%), *SOS1* (1.3%), *PDGFRA* (1.3%), *ERBB2* (1.3), and *BRAF* (1.3%). Importantly, no acquired mutations in the selected list of genes were identified in the control cohort of patients with pre- and post-chemotherapy tumor genomic profiling (Fig 2B). Similarly, acquired mutations in *STK11*, *KEAP1*, *B2M*, *APC*, *CDKN2A*/*B*, *JAK1*, *NF1*/*2, MAP3K1*, *MAP2K4, PDGFRA*, and *ERBB2* were not detected among patients with AR to EGFR tyrosine kinase inhibitor therapy (N = 72), as well as among patients with AR to ALK (N = 8), MET (N = 4), RET (N = 2), ROS1 (N = 2), and BRAF (N = 1) inhibitor therapy, further suggesting that acquired mutations in these genes are more likely to develop as specific mechanisms of resistance to ICI. As expected, among patients with intervening targeted therapies, we found acquired mutations in *EGFR* (19.1%), *PIK3CA* (7.8%), *KRAS* (3.3%), and *BRAF* (2.2%), which are known resistance mechanisms to these therapies in NSCLC^12^ (Fig 2C). The distribution of acquired genomic changes in patients receiving intervening chemotherapy or targeted therapy is shown in the Data Supplement (Fig S8).

We next examined acquired CNAs in each of these three cohorts. Among patients treated with ICI, the most common acquired heterozygous losses included *B2M* (22.8%), *STK11* (17.7%), *SMARCA4* (17.7%), and *KEAP1* (16.5%). Additional acquired heterozygous deletions included *PTEN* (10.1%), *CDKN2A*/*B* (10.1%), *CD274*/*PDCD1LG2* (8.9%), *JAK2* (7.6%), *FBXW7* (7.6%), and *JAK1* (6.3%). We also identified homozygous deletion in *CDKN2A*/*B* (8.9%), *NF1*/*2* (2.5%), *CD274*/*PDCD1LG2* (1.3%), and *JAK2* (1.3%; Fig 2D). Of note, acquired heterozygous deletions with concurrent acquired mutations were found in *STK11* (5.1%), *SMARCA4* (2.5%), *KEAP1* (1.3%), *B2M* (1.3%), and *JAK1* (1.3%), indicating biallelic inactivation of these genes (Fig 1). Among gene-level copy gains, acquired high-level amplification was found in *MDM2* (5.1%), *KRAS* (3.8%), *MYC* (2.5%), *AKT1* (1.3%), and *EGFR* (1.3%) in ICI-resistant samples (Fig 2D). Among patients with AR to chemotherapy or targeted therapies, we also noted similar acquired CNA patterns (Figs 2E and 2F). However, no biallelic losses in *STK11*, *KEAP1*, *SMARCA4*, *B2M*, *JAK1*, and *CD274*/*PDCD1LG2* were noted in these control cohorts.

Because 25% of patients who developed AR to ICI in our cohort also received at least one additional line of therapy between the pre- and post-ICI tumor biopsy, we also examined acquired genomic changes in a third control cohort of patients (N = 17) who received multiple lines of therapy including both chemotherapy and targeted therapy (but not immunotherapy) between tumor biopsies. Similarly, in this cohort, we only identified acquired *EGFR* and *ERBB2* mutations, which were developed as resistance mechanisms to targeted therapy (Data Supplement, Figs S9A-S9B). No acquired mutations in *STK11*, *KEAP1*, *B2M*, *JAK1*, and *APC* were noted. In examining CNAs in this cohort, we again noted similar patterns of heterozygous deletions as was observed in the immunotherapy cohort and in the control groups of patients with tumor genomic profiling performed before and after chemotherapy or targeted therapy (Data Supplement, Fig S9C). A list of the baseline and acquired genomic changes identified at the time of AR to ICI is shown in the Data Supplement (Tables S5 and S6).

We next examined whether there was a difference in the frequency of acquired genomic alterations according to the time to developing resistance to ICI (3-6 months *v* ≥6 months^13^). In all-comers treated with ICI, there was no difference in the frequency of acquired genomic alterations between patients who developed AR within 6 months of ICI initiation and those who developed AR ≥6 months of treatment (Data Supplement, Figs S10A-S10C). However, among patients who received PD-(L)1 monotherapy, the frequency of acquired mutations at the time of AR was significantly higher in cases who developed resistance ≥6 months after ICI initiation versus those who developed AR within the first 6 months of treatment (58.3% *v* 10.5%, *P* < .01; Data Supplement, Figs S11A-S11C), possibly reflecting the emergence of resistant clones after prolonged exposure to PD-1 monotherapy. By contrast, there was no difference in the frequency of acquired mutations and CNAs according to the time to AR among patients who developed resistance to chemoimmunotherapy (Data Supplement, Figs S11D-S11F). Subgroup analyses showing the frequency of acquired genomic changes by best response to ICI ± chemotherapy, line of therapy, and PD-L1 expression levels (<1%, 1%-49%, and ≥50%) are shown in the Data Supplement (Fig S12).

In this cohort, there was no difference in the median TMB or in the median aneuploidy score (Data Supplement, Figs S13A-S13B) between pre- and post-ICI samples, suggesting that acquired mutations and CNAs were not simply the result of increased mutational load and tumor aneuploidy. Similarly, no differences in TMB or aneuploidy were noted in the two control cohorts (Data Supplement, Fig S13). A swimmer's plot summarizing time to AR to ICIs, length of time on therapy for individual patients, and key acquired mutations at AR is shown in the Data Supplement (Fig S14).

### Immunophenotypic Changes in NSCLC With AR to PD-(L)1 Blockade

**FIG 3.**
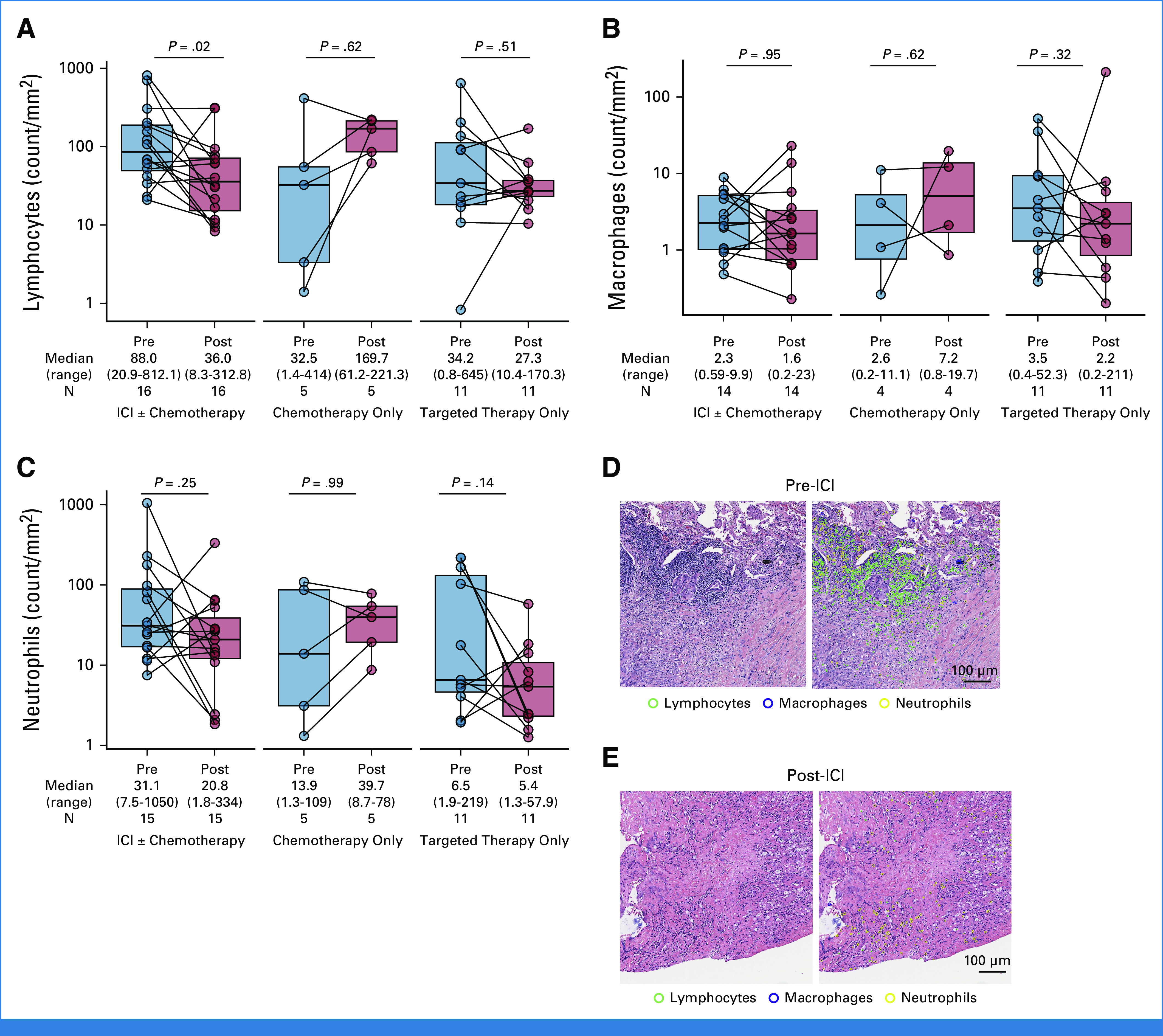
Paired box plots showing the change in the density of tumor-infiltrating (A) lymphocytes, (B) macrophages, and (C) neutrophils among patients with matched H&E-stained slides before versus after intervening immunotherapy, chemotherapy, or targeted therapy. Immune cell density was quantified on digital pathology imaging using the validated machine learning algorithm PathML. Representative images of (D) a pre-ICI sample and (E) a matched postimmunotherapy tumor sample that underwent immune cell subset deconvolution using PathML are shown, reflecting a significant decrease in intratumoral lymphocytes in the immunotherapy resistant sample. Groups were compared using the paired Wilcoxon signed-rank test. H&E, hematoxylin and eosin; ICI, immune checkpoint inhibitor.

We next sought to determine whether there were changes in the tumor immunophenotype after development of AR to ICI in NSCLC. Of the 82 patients who developed AR, 16 had matched pre- and post-ICI digitalized hematoxylin and eosin–stained slides that passed quality metrics. We used our validated ML approach to quantify immune cell types (PathML^11^; Data Supplement, Fig S15). We noted that the density of tumor-infiltrating lymphocytes (TILs) significantly decreased after treatment with ICI (median 88 *v* 36 cells/mm^2^, *P* = .02, Fig 3A). By contrast, there was no difference in TIL density among patients with pre- and post-chemotherapy (*P* = .62) and pre- and post-targeted therapy (*P* = .51) tumor samples (Fig 3A). There was no difference in tumor-infiltrating macrophages or neutrophils before versus after immunotherapy, chemotherapy, or targeted therapy (Figs 3B and 3C). A representative case of pre- and post-ICI tumor biopsy showing a marked decrease in TILs as assessed by PathML at the time of AR to immunotherapy is shown in Figures 3D-3E.

**FIG 4.**
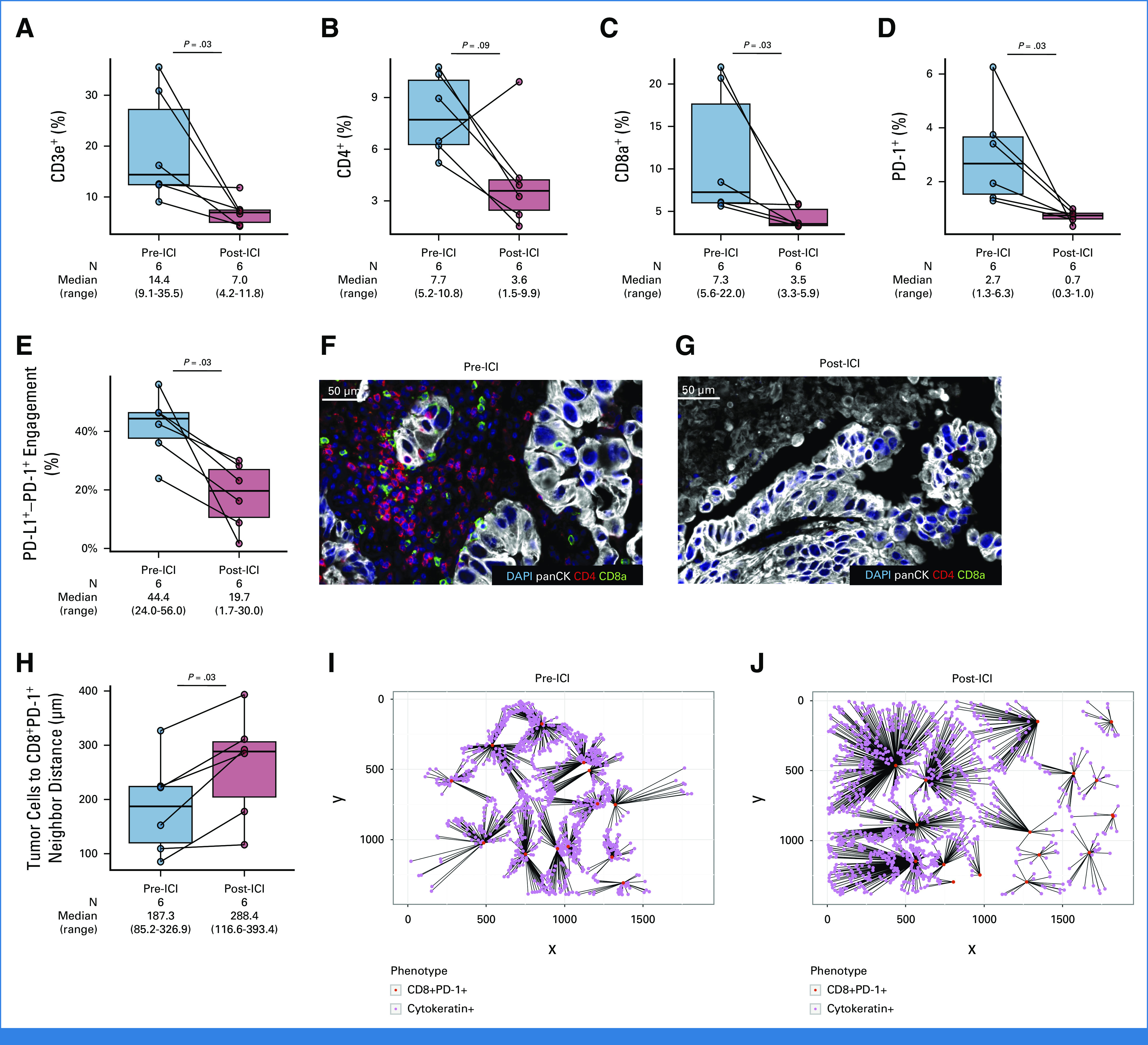
Paired box plots showing changes in (A) CD3e^+^, (B) CD4^+^, (C) CD8a^+^, and (D) PD-1^+^ T cells in patients with pre- and post-ICI tumor samples that underwent multiplexed immunofluorescence. (E) Paired box plot showing a significant decrease in PD-1–PD-L1 engagement at the time of AR to ICI. Representative case of a patient with (F) pre- and (G) post-immunotherapy multiplexed immunofluorescence showing significant decrease in tumor-infiltrating CD4 and CD8a T cells. (H) Paired box plots showing significant increase in the neighbor distance between tumor cells (cytokeratin+) and CD8^+^PD-1^+^ T cells at the time of AR to immunotherapy. Representative ray plots showing increase in the neighbor distance between tumor cells (cytokeratin+) and CD8^+^PD-1^+^ T cells between (I) pre- and (J) post-ICI tumor samples. AR, acquired resistance; ICI, immune checkpoint inhibitor.

To further characterize the immunophenotypic changes in NSCLC with AR to ICI, we next performed mIF to assess 21 different markers (Supplementary Methods) on tumors from six patients with tissue available from pre- and post-ICI samples. We noted a significant decrease in tumor-infiltrating CD3e^+^ T cells (*P* = .03), CD8a^+^ T cells (*P* = .03), and PD-1^+^ cells (*P* = .03) in post-ICI samples, compared with pre-ICI samples (Fig 4). There was also a significant decrease in CD3e^+^PD-1^+^, CD8a^+^PD-1^+^, and CD4^+^PD-1^+^ T cells, as well as CD45^+^ cells in post- versus pre-ICI tumor samples (*P* < .05, Data Supplement, Fig S16). There was no difference in the density of T regulatory cells, M1/M2 macrophages (CD68^+^, CD163^+^, CD206^+^), CD11^+^, TCF1^+^, and SMA^+^ cells (Data Supplement, Fig S16), as well as in PD-L1 expression on tumor cells and nontumor cells, and in total between pre- and post-ICI samples as assessed by mIF (Data Supplement, Fig S17), or in PD-L1 expression as assessed by IHC (Data Supplement, Fig S18). Spatial immunophenotyping showed a significant decrease in PD-1–PD-L1 engagement by mIF at the time of AR (*P* = .03, Fig 4E, Data Supplement, Fig S19). Representative images of pre- and post-ICI samples that underwent mIF are shown in Figures 4F and 4G. Finally, we noted a significant increase in the neighbor distance between tumor cells and CD8+PD-1+ T cells (*P* = .03, Fig 4H). Ray plots showing increased tumor cells to CD8^+^PD-1^+^ T cells distance at AR are shown in Figures 4I and 4L. A detailed summary of immunophenotypic changes identified at the time of AR is reported in the Data Supplement (Table S7).

### HLA Class I Expression at the Time of AR to PD-(L)1–Based Therapies

**FIG 5.**
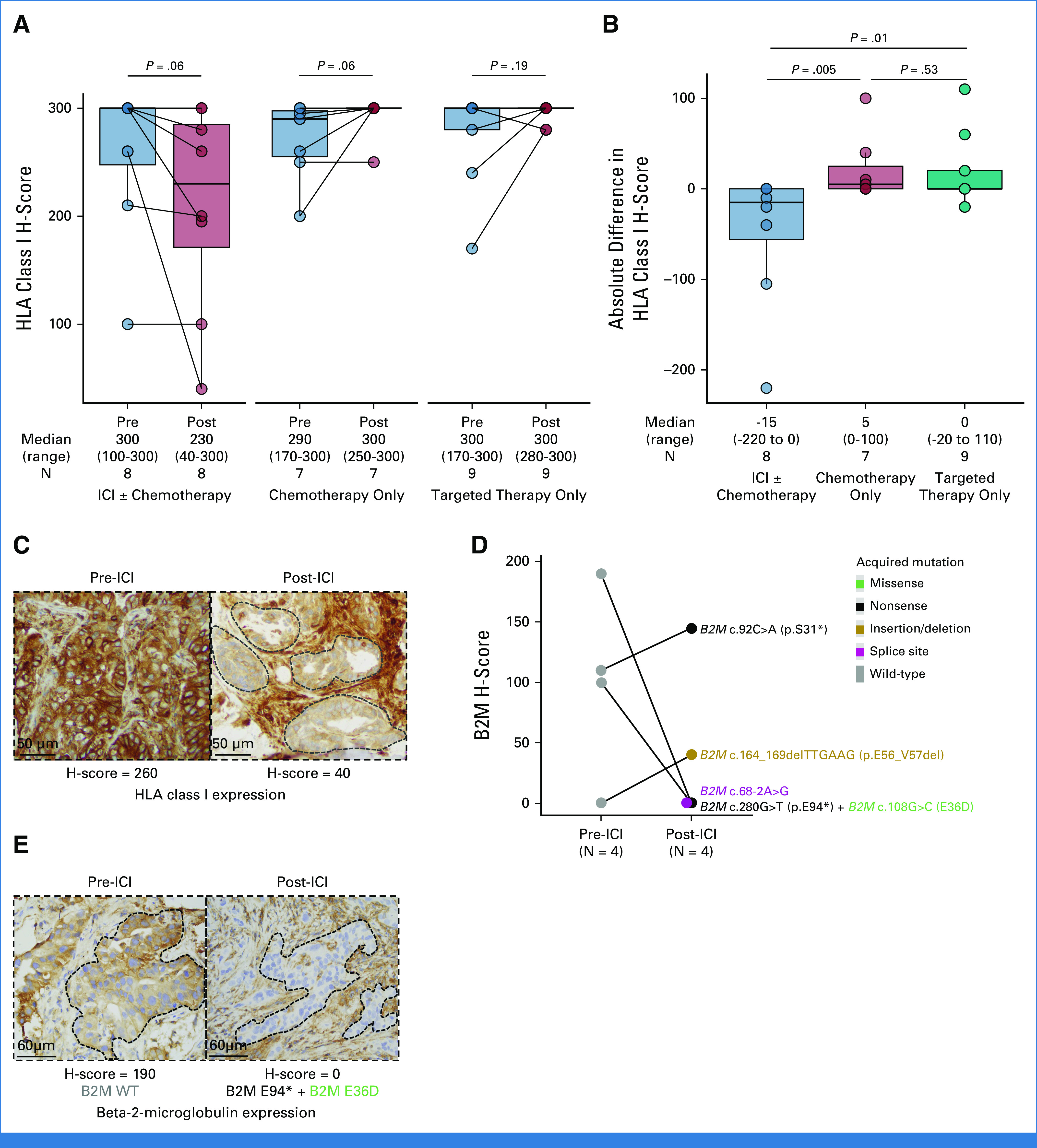
(A) Paired box plot showing absolute changes in HLA class I H-score among patients with pre- and post-ICI, chemotherapy, and targeted therapy tumor samples that underwent HLA class I IHC. (B) Percent change in HLA class I H-score among patients with pre- and post-ICI, chemotherapy, and targeted therapy tumor samples that underwent HLA class I IHC. (C) Representative case of pre- and post-ICI tumor biopsy showing significant decrease in HLA class I expression at the time of acquired resistance. (D) Change in B2M expression by H-score between pre- and post-ICI tumor samples. Individual acquired mutations are also reported. (E) Representative case of decreased B2M expression by IHC in a patient with acquired *B2M* nonsense mutation (c.280G>T; p.E94*) with concurrent *B2M* missense mutation (c.108G>C; E36D). Groups were compared using the paired Wilcoxon signed-rank test. B2M, beta-2-microglobulin; ICI, immune checkpoint inhibitor; IHC, immunohistochemistry.

Increased HLA class I expression correlates with improved outcomes to ICI across cancer types.^14^ However, whether changes in HLA class I expression mediate the development of AR to ICI in lung cancer is unknown. To determine whether changes in HLA class I expression contribute to the development of AR to ICI, we performed pan-HLA class I IHC on NSCLC samples from patients with available pre- and post-ICI tumor tissue (N = 8), as well as in the two control cohorts of patients with pre- and post-chemotherapy and targeted therapy with available tissue. We found that NSCLC samples collected at the time of AR had a marked decrease in HLA class I expression compared with pre-ICI samples (median H score: 230 *v* 300, *P* = .06). By contrast, there was a slight increase in HLA class I expression between pre- and post-chemotherapy (N = 7, *P* = .06) and pre- and post-targeted therapy (N = 9, *P* = .19) NSCLC samples (Fig 5A). When comparing the difference in HLA class I H scores, there was a significant drop in HLA class I expression in the immunotherapy cohort at the time of AR compared with the chemotherapy (*P* = .005) and the targeted therapy (*P* = .01) cohorts (Fig 5B). Figure 5C illustrates a representative case with a marked decrease HLA class I expression in an ICI-resistant sample compared with baseline.

We finalty tested whether the acquired mutations in the *B2M* gene found at the time of AR to ICI were associated with changes in B2M expression. Among the five patients with acquired *B2M* mutations, four had matched pre- and post-ICI tissue available for IHC. Two patients (50%) with acquired *B2M* mutations including a nonsense + missense mutations (p.E94* + p.E36D) and splice site mutation (c.68-2A>G) had abolished B2M expression by H-score (190 to 0 and 100 to 0, respectively; Figs 5D and 5E). In the remaining two cases (*B2M* in-frame deletion [p.E56_V57del], and *B2M* nonsense mutation [p.S31*]), there was mild increase in B2M expression by H-score (110 to 145 and 0 to 40, respectively; Fig 5D).

## DISCUSSION

In this study, we examined matched pre- and post-ICI samples from patients who developed acquired resistance to ICI. We identified recurrent acquired genomic changes, decreased TILs and HLA class I expression in tumor biopsies at disease progression on ICI, not seen in patients treated with chemotherapy or targeted therapies.

Previous studies have shown that loss-of-function mutations in *STK11*, *KEAP1*, and *SMARCA4* drive primary resistance to ICI in lung cancer.^15,16^ Here, we demonstrate that these alterations can also mediate AR to ICI. This is significant as these mutations create vulnerabilities exploitable by novel therapies to restore ICI sensitivity.^17-20^

Additionally, 6.3% of patients acquired *B2M* mutations, which resulted in abolished B2M expression by IHC in 50% of evaluable cases. *B2M* alterations have previously been reported in nonresponders compared with responders to PD-1 and CTLA-4 inhibitors among patients with melanoma, and to associate with reduced B2M expression.^21^ In a previous analysis of 14 ICI-resistant NSCLC samples, an acquired homozygous deletion of B2M that caused lack of cell-surface HLA class I expression in the tumor and a matched patient-derived xenograft was identified, and CRISPR-mediated knockout of *B2M* in an immunocompetent lung cancer mouse model conferred resistance to PD-1 blockade in vivo, confirming the role of B2M loss in resistance to ICIs.^3^ Although our results are also supportive of mechanistic role of B2M loss in mediating AR to ICI, these findings should be interpreted with caution, given the small sample size.

Additionally, we found an acquired biallelic loss of *JAK1*, as well as an acquired homozygous deletion in *JAK2* in two different patients. JAK1 and JAK2 are essential signal transducers of the INFγ pathway, therefore, in the setting of AR to ICI, a tumor may become insensitive to INFγ because of loss of JAK1/2.^22^ Consistently, previous evidence from whole-exome sequencing of four ICI-resistant melanomas revealed acquired loss in *JAK1* and *JAK2*, and preclinical modeling of *JAK1* and *JAK2* truncating mutations also resulted in cancer cells insensitivity to INFγ, supporting a mechanistic role of these mutations in mediating resistance to ICI.^4^

Other acquired genomic alterations previously associated with an impaired antitumor immune response through the activation of the MAPK, PI3K/Akt/mTOR, and wingless type/β-catenin pathways^23-26^ were observed among patients with AR to ICI but not among those who were treated with chemotherapy. These included activating mutations in *PIK3CA*, *SOS1*, *ERBB2*, and *BRAF*, and loss-of-function mutations in *NF1*/*NF2* and *APC*. Importantly, drugs targeting these pathways are either FDA approved or under investigation, and preclinical evidence has shown that PI3K/Akt/mTOR and MAPK inhibition may synergize with immunotherapies to resensitize resistant tumors to ICI. Nonetheless, these results remain exploratory and preclinical validation is necessary.

Finally, we demonstrated decreased TILs and HLA class I expression at the time of AR to ICI. Reduced HLA expression has been recognized as a mechanism of escape from antitumor immunity,^27^ and the most common mechanisms of HLA-I losses are reversible defects that can be pharmacologically exploited to restore HLA expression.^27^

Limitations of this study include the heterogeneity of our cohort (ICI ± chemotherapy) and the small sample size of some subgroups (HLA IHC, and mIF). Although small (25%), a fraction of patients received another line of therapy in addition to ICI between tumor biopsies. Nonetheless, we included a third control cohort of patients who received multiple lines of therapy including both chemotherapy and targeted therapy between tumor biopsies, which addresses this limitation. Another limitation is the inclusion of patients who developed AR after ≥3 months of ICI therapy. We used this threshold to reflect the enrollment of patients treated with ≥second-line ICI in this historical cohort, in which the median PFS from ICI is <6 months.^28-30^

In conclusion, mechanisms of AR to ICI are heterogeneous and require personalized post-ICI strategies.
